# Evaluation of the clinical impact of the differences between planned and delivered dose in prostate cancer radiotherapy based on CT‐on‐rails IGRT and patient‐reported outcome scores

**DOI:** 10.1002/acm2.13780

**Published:** 2022-09-10

**Authors:** Jacob Hammers, Daniel Lindsay, Ganesh Narayanasamy, Shivani Sud, Xianming Tan, John Dooley, Lawrence B. Marks, Ronald C. Chen, Shiva K. Das, Panayiotis Mavroidis

**Affiliations:** ^1^ Department of Radiation Oncology University of North Carolina at Chapel Hill North Carolina USA; ^2^ Department of Radiation Oncology University of Arkansas for Medical Sciences Arkansas USA; ^3^ Lineberger Comprehensive Cancer Center University of North Carolina Hospitals Chapel Hill North Carolina USA

**Keywords:** image‐guided radiotherapy, normal tissue complication probability, prostate radiotherapy, radiotherapy outcome, relative seriality

## Abstract

**Purpose:**

To estimate the clinical impact of differences between delivered and planned dose using dose metrics and normal tissue complication probability (NTCP) modeling.

**Methods:**

Forty‐six consecutive patients with prostate adenocarcinoma between 2010 and 2015 treated with intensity‐modulated radiation therapy (IMRT) and who had undergone computed tomography on rails imaging were included. Delivered doses to bladder and rectum were estimated using a contour‐based deformable image registration method. The bladder and rectum NTCP were calculated using dose–response parameters applied to planned and delivered dose distributions. Seven urinary and gastrointestinal symptoms were prospectively collected using the validated prostate cancer symptom indices patient reported outcome (PRO) at pre‐treatment, weekly treatment, and post‐treatment follow‐up visits. Correlations between planned and delivered doses against PRO were evaluated in this study.

**Results:**

Planned mean doses to bladder and rectum were 44.9 ± 13.6 Gy and 42.8 ± 7.3 Gy, while delivered doses were 46.1 ± 13.4 Gy and 41.3 ± 8.7 Gy, respectively. *D*_10cc_ for rectum was 64.1 ± 7.6 Gy for planned and 60.1 ± 9.3 Gy for delivered doses. NTCP values of treatment plan were 22.3% ± 8.4% and 12.6% ± 5.9%, while those for delivered doses were 23.2% ± 8.4% and 9.9% ± 8.3% for bladder and rectum, respectively. Seven of 25 patients with follow‐up data showed urinary complications (28%) and three had rectal complications (12%). Correlations of NTCP values of planned and delivered doses with PRO follow‐up data were random for bladder and moderate for rectum (0.68 and 0.67, respectively).

**Conclusion:**

Sensitivity of bladder to clinical variations of dose accumulation indicates that an automated solution based on a DIR that considers inter‐fractional organ deformation could recommend intervention. This is intended to achieve additional rectum sparing in cases that indicate higher than expected dose accumulation early during patient treatment in order to prevent acute severity of bowel symptoms.

## 1 INTRODUCTION

With the widespread implementation of image guided radiotherapy (IGRT), the standard of care for the treatment of prostate cancer using radiotherapy incorporates fractional image guidance to improve conformity between the planned dose and the delivered dose to the regions of interest.^1^, ^2^, ^3^, ^4^, ^5^, ^6^ The high boundary dose gradients created to spare the organs‐at‐risk (OAR) require robust verification of dose delivery and quality assurance of the inverse treatment planning process.^5^, ^7^, ^8^, ^9^, ^10^, ^11^, ^12^, ^13^ Variable filling status of bladder and rectum leads to high inter‐fractional variation in the size of the organs throughout treatment causing less consistent dose delivery.

Recent studies have indicated the rapid development and verification of deformable image registration (DIR) algorithms for use in adaptive radiotherapy (ART).^14^, ^15^, ^16^, ^17^, ^18^, ^19^, ^20^, ^21^, ^22^, ^23^ The most reliable DIR algorithms currently in use rely on automated intensity‐based spatial information of a reference and target image, manual geometric contour‐based segmentation information from physician guidance, or a hybrid semi‐automated model incorporating both intensity‐based spatial information and geometric contour‐based segmentation information.^24^ In order for ART to be most effective, consideration must be given to the specific anatomy for which each fraction of treatment was administered.

It is well established that the impact of intensity‐modulated radiation therapy (IMRT) is reflected on patient‐reported outcome (PRO) data (such as those reported by the prostate cancer symptom indices (PCSI)).^25^, ^26^, ^27^, ^28^, ^29^, ^30^, ^31^, ^32^ However, the association of dose accumulated in the bladder and rectum in a manner that explicitly considers inter‐fractional organ deformation and PRO is not well‐studied.^26^, ^27^, ^28^, ^29^, ^30^, ^31^, ^32^ The association of PRO–PCSI data of urinary or rectal dysfunction with accumulated dose in the bladder and rectum while considering anatomical changes during treatment, DIR, and a radiobiological model were employed here. As DIR enables examination of inter‐fractional variation in local anatomy resulting from changes in patient position, alignment, and organ deformation, normal tissue complication probability (NTCP) models provide the basis for a clinically related comparison of the planned and delivered doses in the regions of interest.^5^, ^6^, ^8^, ^10^, ^13^, ^16^, ^18^, ^19^, ^20^, ^21^, ^22^, ^23^, ^24^, ^26^, ^33^ The relative seriality model enables the calculation of the radiobiologically effective uniform dose ($\bar{\bar{D}}$) and NTCP from the dose volume histogram (DVH).^26^, ^33^, ^34^, ^35^, ^36^, ^37^, ^38^, ^39^, ^40^

In this work, we present a statistical analysis examining the association between PRO related to PCSI and delivered dose considering the fractional patient setup and organ deformation uncertainties. Understanding of the association between PRO and dosimetric parameters derived from the DVH and NTCP model provides enhanced capability to perform ART and estimate the clinical effect that local organ deformation may have on the treatment plan and outcome.

## 2 METHODS

### 2.1 Study cohort

A total of 46 of consecutive prostate cancer patients, who received IMRT from 2010 to 2015 at the University of North Carolina at Chapel Hill were included in this IRB‐approved analysis. Prostate bed radiation was given for patients after radical prostatectomy with either adverse pathological features (adjuvant IMRT) or with biochemical recurrence (salvage IMRT). Patients received 40–44 fractions of 1.8 Gy/fraction to the target. The treatment planning system used was PLUNC and the dose calculation algorithm was the Collapse Cone. Nine step and shoot fields were used to produce the IMRT plans. The planning CT images consisted of 512 × 512 pixel slices with voxel dimensions of 1.25 mm × 1.25 mm × 1.5 mm. The dose grid resolution was 3 mm in all the directions. The CTOR images consisted of 512 × 512 pixel slices with voxel dimensions of 1 mm × 1 mm × 3 mm. The dose constraints for bladder and rectum during plan optimization were: bladder average dose (*D*_mean_) < 55 Gy and rectal *D*_10cc_ < 70 Gy. The dosimetric metrics that were used in this analysis are the ones that have commonly been used in our clinic and in the literature (*D*_mean_, *D*_1cc,_ and *V*_50_ for bladder and *D*_mean_, *D*_10cc,_ and *D*_1cc_ for rectum).^41^, ^42^, ^43^, ^44^, ^45^ Androgen deprivation therapy (ADT) consisting of primarily gonadotropin‐releasing hormone (GnRH) agonist (e.g., leuprolide acetate) was used per physician discretion based on disease and patient characteristics.

### 2.2 Image guidance

All patients received image guidance using CT‐on‐rails (CTOR) and were treated with IMRT on a Siemens Artiste linac (Siemens Medical Systems, Concord, CA). The CTOR images consisted of 512 × 512 pixel slices with a field of view of 50 to 100 pixels and voxel dimensions of 1 mm × 1 mm × 3 mm.

### 2.3 Symptom assessment

**FIGURE 1:**
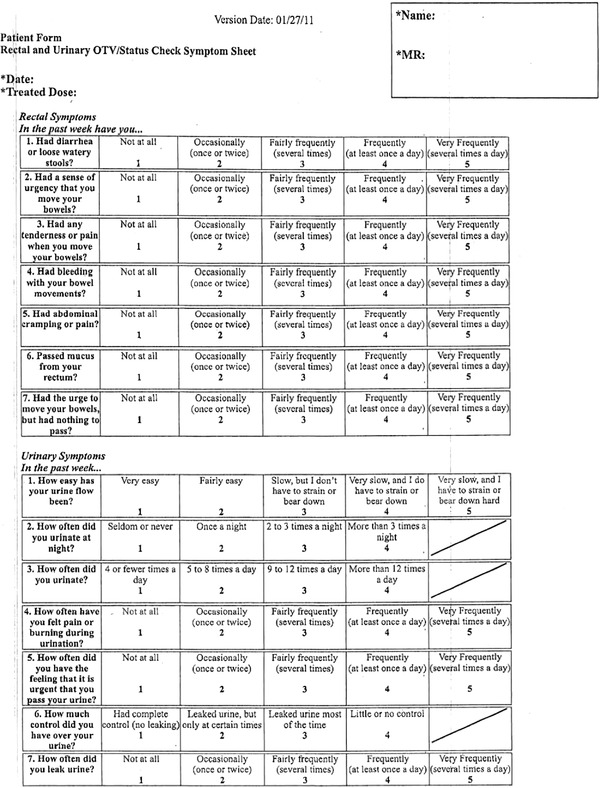
Patient survey for recording PRO. PRO, patient reported outcome

PROs were assessed prospectively using the urinary obstruction/irritation and bowel problems scales of the validated PCSI.^29^ This is a well‐published instrument to assess urinary and bowel symptoms related to prostate cancer treatments. ^25^, ^26^, ^27^, ^28^ At our institution PROs are prospectively assessed for each patient at every visit as part of routine care, including prior to treatment start (baseline), weekly on‐treatment‐visits, and post‐treatment follow‐up visits. Fourteen rectal and urinary symptoms are assessed by PROs such as diarrhea, proctitis, urinary frequency, urinary incontinence, urinary retention, urinary tract pain and urinary urgency—allowing direct comparison. PCSI questions (refer Figure 1) are rated on a Likert scale of 1 to 4 or 5 with higher numbers indicating greater severity of a given symptom. Based on previously validated cut‐offs for this instrument, we dichotomized responses such that an increase by ≥2 levels in a symptom at 12 months post‐RT compared to baseline was considered clinically meaningful.^26^ More specifically, we collected the individual patient scores at 12 months post‐RT and we subtracted them from the corresponding scores at baseline. If the net difference was ≥2 levels then we characterized that patient as a responder regarding this symptom, otherwise the patient was characterized as a non‐responder. In the present analysis, the individual patient scores were used.

### 2.4 Accumulated dose estimation

**FIGURE 2:**
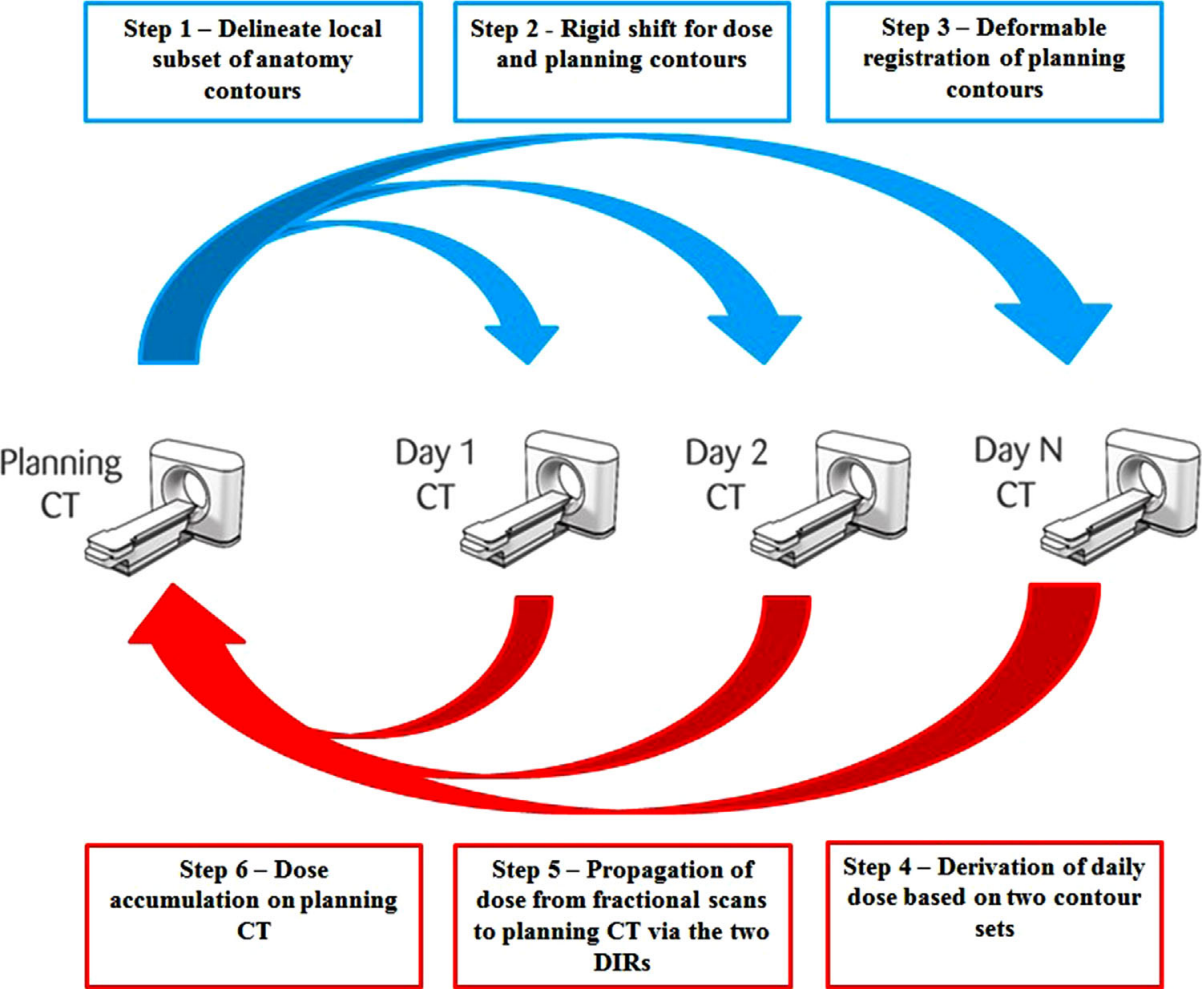
Graphical representation of the dose registration and accumulation procedure, which was applied on each OAR. Image guidance was generated with CT‐on‐rails and the patients were treated with IMRT. OAR, organs at risk; CT, computed tomography; IMRT, intensity‐modulated radiation therapy

The delivered dose distributions to bladder and rectum were calculated using a previously validated DIR algorithm (hybrid intensity–contour based referred as “contour‐based”) and were compared with the respective planned doses.^43^ Figure 2 shows a depiction of the dose accumulation process. The planning CT was used as a reference to accumulate dose based on the applied DIR algorithm in weekly accumulation sets. In this process, image data were imported to MIM version 6.8 beta (MIM Software, Inc., Cleveland, OH). The contours of bladder and rectum were delineated by a radiation oncologist on weekly IGRT scans (Step 1). A rigid image registration was performed between the planning CT and each fractional IGRT scan separately. The planned dose matrix was registered to each IGRT using the corresponding rigid registration (rigid shift) (Step 2). A contour‐based DIR was performed between the planned contours of bladder and rectum and the corresponding contours drawn on each IGRT (Step 3). For each IGRT, the dose matrices of bladder and rectum were extracted by the complete dose matrix (Step 4). Using the deformation vector fields derived from each DIR on the extracted bladder and rectum dose matrices, we managed to register the IGRT dose distributions to those two structures back to the planning CT from each IGRT (deformable registration this time). Those dose matrices were also scaled to a fractional dose level (Step 5). All those fractional dose matrices of bladder and rectum could now be summed up and produced to cumulative delivered dose matrices (Step 6) from which the corresponding organ DVHs and dose‐volume metrics were calculated.

### 2.5 Relative seriality model (s model)

For the calculation of the NTCP values, the relative seriality NTCP model was used. The relative seriality model is a radiobiological model that estimates the normal tissue probability of injury (*P*_I_) based on a cell survival functional model:

(1) $$P_{I}\left(D,V\right)={\left[1-{\left(1-P_{I}{\left(D,V_{\mathrm{ref}}\right)}^{s}\right)}^{V/V_{\mathrm{ref}}}\right]}^{1/s}$$

where *D* is the uniform dose irradiating normal tissue volume *V* as a function of reference volume *V*_ref_ for which the values of *D*_50_and *γ* were calculated. *D*_50_ is the dose which gives a response probability of 50% and *γ* is the maximum normalized value of the dose‐response gradient.^33^, ^34^, ^35^, ^36^, ^37^, ^38^, ^39^, ^40^ The normal tissue response to a heterogeneous dose distribution is given by:

(2) $$P_{I}\left(\vec{D},\vec{V}\right)={\left[1-\prod_{i=1}^{M}{\left(1-P{\left(D_{i},V_{ref}\right)}^{s}\right)}^{\Delta v_{i}}\right]}^{1/s}$$

where $\Delta v_{i}$ ( =$\Delta V_{i}/V_{\mathrm{ref}}$ ) is the fractional irradiated sub‐volume of volume *V_i_* compared to *V*_ref_, and *s* is the relative seriality parameter that characterizes the serial organization of the functional subunits of the organ. *P*(*D_i_*) is the response probability of a given voxel and is defined by the following mathematical expression:^33^, ^34^, ^35^, ^36^, ^37^, ^38^, ^39^, ^40^

(3) $$P\left(D\right)=\exp\left\{-e^{e\gamma -\left(D/D_{50}\right)\cdot \left(e\gamma -\ln\ln2\right)}\right\}$$

$\bar{\bar{D}}$ is the biologically effective uniform dose, and it is defined as the dose that causes the same NTCP as the 3‐dimensional dose (planned or delivered) and is averaged over dose distribution and organ radio‐sensitivity. $\bar{\bar{D}}$ is derived from the following expression:^33^

(4) $$P_{I}\left(\vec{D},\vec{V}\right)=e^{-e\gamma \left(\bar{\bar{D}}/D_{50}\right).\left(e\gamma -\ln\ln2\right)}$$

**FIGURE 3:**
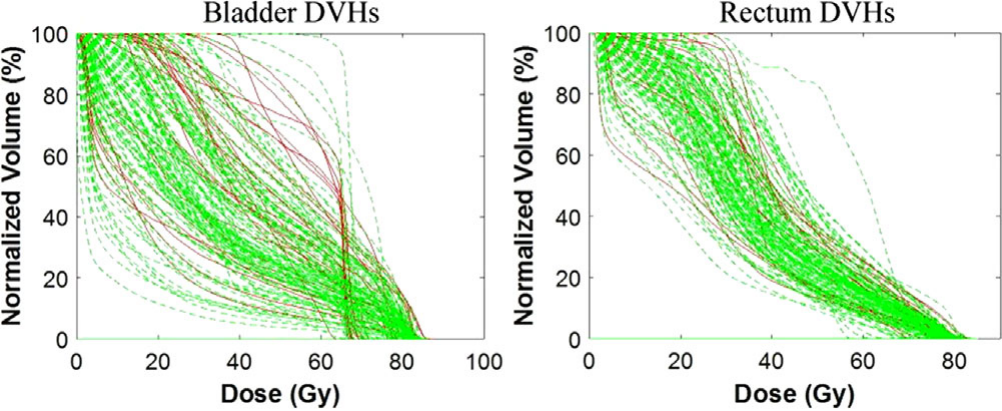
The DVHs of bladder (left) and rectum (right) based on the patient‐reported scoring system. The red lines indicate the patients, who reported the examined symptoms 12‐months post‐treatment. Response was defined as a change in severity (from baseline) of ≥2. DVH, dose volume histogram

Using a larger cohort of 141 patients for which the complete treatment plans and PCSI scores were available, the dose‐response curves of bladder and rectum were determined for two of the urinary and bowel symptoms of PCSI at 12 months post‐RT (Appendix: *u*_2_: frequency of urinating at night; and *r*_1_: diarrhea or loose watery stools), which showed the highest correlation with dose (AUC = 0.65 and 0.61, respectively) compared with the rest of the symptoms. The model parameters describing those dose‐response curves are: *D*_50_ = 80.9 (65.7–108.2) Gy, γ = 0.44 (0.31–0.57), and *s* = 0.0001 (0.00001–0.0007) for bladder and *D*_50_ = 67.0 (64.5–72.0) Gy, γ = 2.5 (0.8–7.1), and *s* = 1.0 (0.7–7.0) for rectum. Figure 3 shows the DVHs of bladder and rectum for the patients with symptoms (in red) and without symptoms (in green).

### 2.6 Statistical analysis

The ability of the NTCP models to distinguish patients with and without the examined symptoms was evaluated using the area under the curve (AUC) measure, which is used as a summary of the ROC curve.^38^ The two‐sample *t*‐test was also applied to compare the NTCP values of the planned and delivered dose distributions for bladder and rectum, respectively (the null hypothesis is that the true mean difference is zero).

## 3 RESULTS

The results of the dosimetric and NTCP comparisons of the 46 patients are shown in Table 1. For the contour‐based DIR, the average NTCP values of the estimated delivered doses to bladder and rectum were higher and lower, respectively, compared to the values calculated from the respective plans. Those findings are in line with the corresponding dosimetric results of *D*_mean_, *D*_1cc_, and *V*_50_ for bladder and *D*_mean_, *D*_1cc_, and *D*_10cc_ for rectum. Based on the results shown in Table A1 (Appendix), the D_mean_ dose constraint of bladder was violated in 37 plans compared to 22 in the corresponding delivered doses. The *D*_10cc_ dose constraint of rectum was violated in 15 plans compared to 14 in the corresponding delivered doses. For bladder, the dose constraint violation was observed in both the planned and delivered dose distributions on nine cases, and in just one case for rectum.

**TABLE 1:** Summary of the NTCP values of bladder and rectum calculated from the planned and estimated delivered doses. The results are broken down per deformable image registration (DIR) algorithm (intensity‐based and contour‐based)

**Organ**	**Bladder**				**Rectum**			
**metrics**	**NTCP (%)**	***D*_mean_ (Gy)**	***D*_1cc_ (Gy)**	***V*_50_ (%)**	**NTCP (%)**	***D*_mean_ (Gy)**	***D*_1cc_ (Gy)**	***D*_10cc_ (Gy)**
Plan	22.3 ± 8.4	44.9 ± 13.6	77.9 ± 7.5	42.7 ± 23.9	12.6 ± 5.9	42.8 ± 7.3	75.0 ± 7.1	64.1 ± 7.6
Delivered	23.2 ± 8.4	46.1 ± 13.4	77.2 ± 8.6	44.8 ± 24.8	9.9 ± 8.3	41.3 ± 8.7	72.1 ± 8.6	60.1 ± 9.2

NTCP, normal tissue complication probability.

**FIGURE 4:**
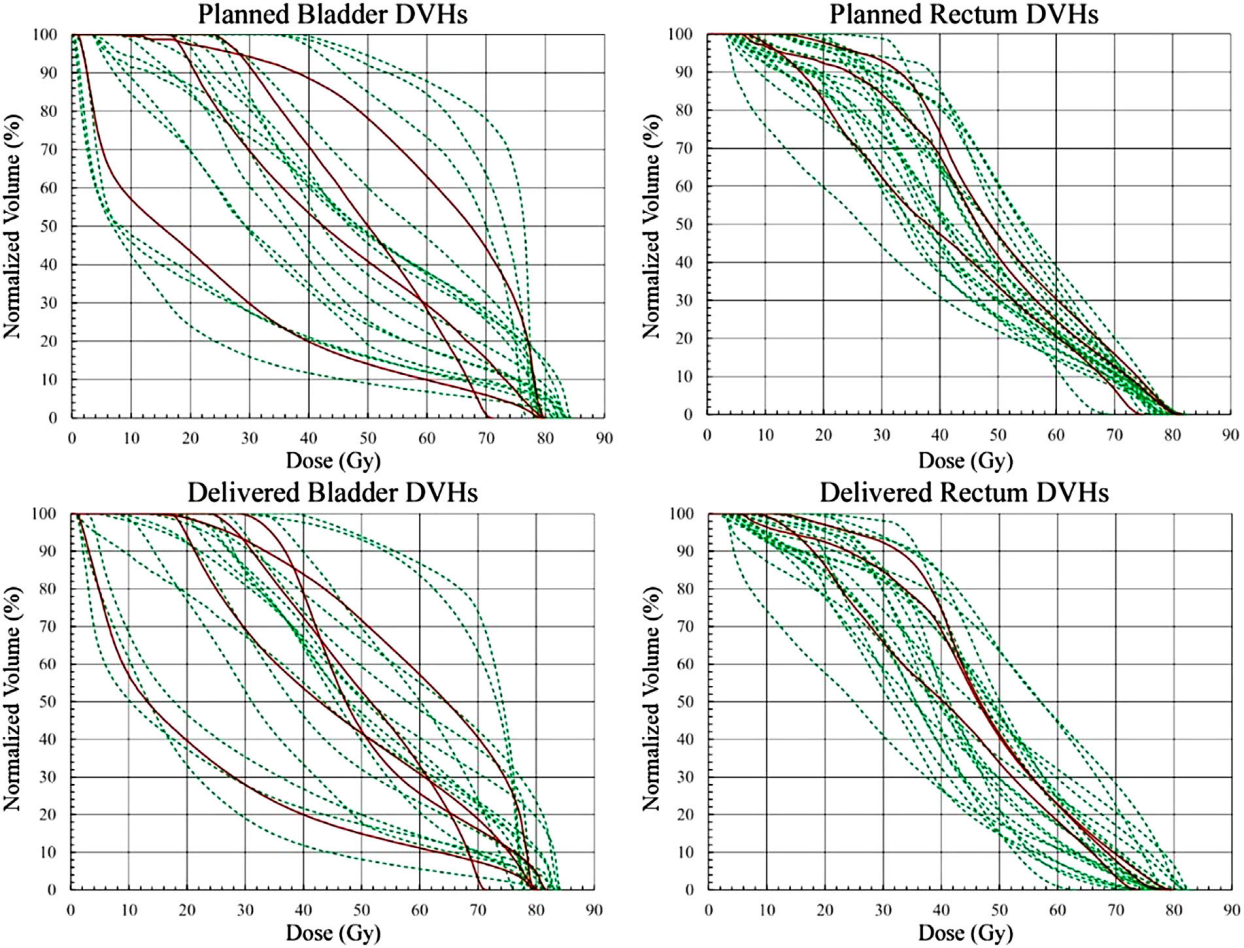
The DVHs of bladder (left) and rectum (right) based on the patient‐reported scoring system. The red lines indicate the patients, who reported the examined symptoms 12‐months post‐treatment. Response was defined as a change in severity (from baseline) of ≥2. DVH, dose volume histogram

Figure 4 shows the DVHs of bladder and rectum for the 25 patients for which both the fractional CTOR scans and PRO data were available and analyzed. Table 2 displays the results of the NTCP for urinary and rectal complications 12 months post‐RT follow‐up times, respectively. Based on those results, the risk for urinary complications is 0.9% higher for the delivered dose distribution compared to the planned doses, whereas the risk for rectal complications is 3.1% lower. For both bladder and rectum, the paired *t*‐test indicated that the planned and delivered NTCP values were different with statistical significance (*p*‐values = 0.03 and 0.01, respectively). However, when the NTCP values are correlated with the PRO, both the planned and delivered doses of bladder show no correlation (AUC = 0.54 and 0.47, respectively), whereas for rectum they show some correlation, which is almost the same for the planned and delivered doses (AUC = 0.68 and 0.67, respectively).

**TABLE 2:** Summary of the NTCP values of bladder and rectum calculated from the planned and estimated delivered doses

**Organ**	**Bladder**			**Rectum**		
**Metric**	**NTCP (%)**	***p*‐Value**	**AUC**	**NTCP (%)**	***p*‐Value**	**AUC**
Planned	22.4 ± 9.2	—	0.54	13.1 ± 5.5	—	0.68
Delivered	23.5 ± 8.3	0.03	0.47	10.0 ± 8.8	0.01	0.67

NTCP, normal tissue complication probability; AUC, area under the curve.

Dosimetric parameters were derived from the plan and contour‐based weekly accumulated DVH, and they are presented in Appendix (Table A1). During treatment planning the following dose constraints were used for bladder and rectum sparing: bladder mean dose < 55 Gy, bladder V_50_ < 55%, and rectum D_10cc_ < 70 Gy. Dosimetric values exceeding the dose constraints are shown in bold. Of the 24 patients exhibiting at least one dosimetric parameter exceeding the dose constraint on either the plan or contour‐based weekly accumulated DVH, PRO data was available for 12, of whom seven reported PCSI complications.

## 4 DISCUSSION

In this work a statistical analysis of the relationship of dosimetric and radiobiological parameters derived from the planned and delivered DVHs with PCSI scores at 12 months post‐treatment is presented. The analysis shows that no statistically significant correlation was found between some dosimetric parameters and PCSI scores at 12 months post‐treatment for both bladder and rectum. Bladder $\bar{\bar{D}}$ and mean dose did not have a significant impact on urinary PCSI scores for any analysis at 12 months post‐treatment.

The relative seriality NTCP model was fitted to the DVHs and the reported PCSI symptoms *u*_2_ and *r*_1_, which are the two symptoms with the highest correlations with dose at 12 months post‐treatment. While mean dose is independent of the NTCP model parameters *D*_50_ and *γ*, and therefore independent of the PCSI symptoms, the radiobiological $\bar{\bar{D}}$ and NTCP indices are functions of *D*_50_ and *γ*. Therefore, while both the planned and delivered rectum NTCP appear to relate to the *r1* PCSI symptoms at 12 months post‐RT, the corresponding bladder NTCP values does not show correlation with the *u2* PCSI symptoms. However, it has to be emphasized that the sample of 25 patients was very small to establish such a correlation in the validation portion of the analysis.

While the best approximation of the ground truth of dose delivered to the OARs during treatment consists of the contour‐based DIR with daily accumulation interval, such an approach demands physician delineation on every patient fraction resulting in a solution which is too impractical and demanding for clinical implementation. However, the use of deep learning or artificial intelligence algorithms may eliminate this requirement in the near future. The dependency of the correlation of dose or NTCP with PCSI symptoms on accurate OAR delineation highlights the need for use of a robust DIR algorithm to estimate inter‐fractional variation in local anatomy for treatment of prostate cancer with IMRT.

The sensitivity of the rectum to clinical variations of dose accumulation indicates that an automated solution based on intensity‐based DIR that considers inter‐fractional organ deformation could recommend intervention intended to spare rectum that experiences higher than expected dose accumulation early during patient treatment in order to prevent acute severity of bowel PCSI symptoms. The comparatively reduced association of dosimetric parameters with severity of PCSI symptoms at 12 months post‐treatment indicates that chronic PCSI symptom expression is less dependent upon inter‐fractional OAR deformations throughout treatment compared to acute, shorter term PCSI symptom expression.

The accuracy of the estimated dose delivered depends on multiple factors including IGRT modality, image scan quality, DIR algorithm, implementation used and so forth. Several publications have studied the importance of role played by the DIR algorithm. In a previous study, our group examined the impact of the uncertainties imposed by the DIR algorithm.^43^

The current study is based on a small number of patients, so its capability to establish such a correlation was limited. However, the workflow that was developed as part of this study can be used to collect the data that are required for such a thorough statistical analysis. Moreover, future examination of PRO should include analysis incorporating reported symptoms from patients treated with VMAT, which was not possible for this study due to the lack of follow up data. Another important future direction should be the correlation of those doses not only with PRO scores post‐RT but also during the course of radiotherapy (acute effects), which seem to have an impact on the quality of life of those patients.

This study ascertains relation between DVH based dosimetric data with patient reported outcome of radiation therapy on prostate cancer, which typically involves multi‐phase treatment plans. Although in clinical practice, DVHs and dose‐volume metrics are commonly used in treatment plan optimization and evaluation, the use of those metrics is not proper for performing dose accumulation. In cases, where the summation of doses from different fractionation schemes, treatment phases or treatment fractions is needed, the proper way to determine the dose delivered to the patient is by summing those doses at voxel level. Voxel‐based strategies for dose accumulation over multiple phases of planning have been reported in the literature.^46^, ^47^ This process may require the performance of high‐quality deformable registration to ensure that the voxels in the different dose matrices correspond to the same voxel in patient's anatomy. After the final delivered dose has been determined, the relevant DVHs and dose‐volume metrics may be calculated and used for plan evaluation and comparison purposes.

## 5 CONCLUSIONS

The average doses, V_50Gy_ and D_1cc_, to bladder were a little higher in the estimated delivered dose distributions than the corresponding planned ones. On the contrary, the average doses, D_10cc_ and D_1cc_, to rectum were systematically lower in the estimated delivered dose distributions than the corresponding planned ones. This pattern was reflected on the corresponding NTCP values of bladder and rectum. The variations in dose delivery due to the fractional patient setup and organ deformation uncertainties indicate that an automated solution based on a DIR that considers inter‐fractional organ deformation could recommend a plan adaptation during the course of the treatment with the intention to achieve additional rectum sparing in the cases where a higher than expected dose accumulation early during patient treatment is observed. In this way, bowel PCSI symptoms post‐RT may be prevented. However, the limited number of patients with complete follow‐up records did not allow for a comprehensive statistical analysis or the establishment of significant correlations between the different dosimetric and radiobiological metrics with the PCSI symptoms at 12 months post‐treatment.

## CONFLICT OF INTEREST

The authors report no conflict of interest in conducting this research.

## AUTHOR CONTRIBUTIONS

Conception of study: Jacob Hammers and Panayiotis Mavroidis. Writing manuscript: Jacob Hammers, Daniel Lindsay, and Panayiotis Mavroidis. Proof‐reading manuscript: Jacob Hammers, Daniel Lindsay, Ganesh Narayanasamy, Shivani Sud, Xianming Tan, John Dooley, Lawrence B. Marks, Ronald C. Chen, and Shiva K. Das.

## Data Availability

Data sharing is not applicable to this article as no new data were created or analyzed in this study.

## Planned and delivered dose metrics against dose constraints

**TABLE A1:** Plan and weekly accumulated dose metrics versus dose constraints. The dosimetric parameters were derived from the plan and contour‐based weekly accumulated DVHs. The dose constraints that were used during treatment planning were bladder mean dose < 55 Gy, bladder *V*_50_ < 55%, and rectum *D*_10cc_ < 70 Gy. Dosimetric values exceeding the dose constraints are shown in bold. Of the 24 patients exhibiting at least one dosimetric parameter exceeding the dose constraint on either the plan or contour‐based weekly accumulated DVH, patient‐reported outcome data were available for 12, of whom seven reported PCSI complications

**Patient**	**Treatment**	**Accumulation algorithm**	**Bladder mean dose (Gy)**	**Bladder V50 (%)**	**Rectum D10cc (Gy)**
**CTOR1**	IMRT	Plan	39.73	25.40	62.11
**CTOR1**	IMRT	Contour‐based	50.89	45.43	60.50
**CTOR2**	IMRT	Plan	48.81	48.05	69.07
**CTOR2**	IMRT	Contour‐based	43.94	41.65	**72.26**
**CTOR3**	IMRT	Plan	53.04	49.36	52.67
**CTOR3**	IMRT	Contour‐based	49.75	43.68	53.32
**CTOR4**	IMRT	Plan	51.44	47.50	69.69
**CTOR4**	IMRT	Contour‐based	**57.19**	**59.32**	55.96
**CTOR5**	IMRT	Plan	42.97	31.44	56.32
**CTOR5**	IMRT	Contour‐based	43.11	32.05	50.10
**CTOR6**	IMRT	Plan	38.21	32.29	**72.98**
**CTOR6**	IMRT	Contour‐based	40.16	34.95	68.04
**CTOR7**	IMRT	Plan	**57.62**	**67.07**	67.19
**CTOR7**	IMRT	Contour‐based	**57.34**	**66.18**	66.22
**CTOR8**	IMRT	Plan	**66.60**	**97.26**	63.63
**CTOR8**	IMRT	Contour‐based	**66.37**	**96.86**	63.31
**CTOR9**	IMRT	Plan	**65.68**	**93.61**	67.00
**CTOR9**	IMRT	Contour‐based	**65.81**	**94.16**	66.52
**CTOR10**	IMRT	Plan	51.53	45.52	70.00
**CTOR10**	IMRT	Contour‐based	54.46	51.45	64.37
**CTOR11**	IMRT	Plan	47.47	48.29	67.04
**CTOR11**	IMRT	Contour‐based	50.06	50.70	63.67
**CTOR12**	IMRT	Plan	**62.15**	**78.12**	65.84
**CTOR12**	IMRT	Contour‐based	**60.15**	**71.65**	62.21
**CTOR13**	IMRT	Plan	45.21	40.86	66
**CTOR13**	IMRT	Contour‐based	45.97	41.6	52.72
**CTOR14**	IMRT	Plan	34.9	24.34	68.41
**CTOR14**	IMRT	Contour‐based	46.89	39.57	**73.84**
**CTOR15**	IMRT	Plan	53.71	54.13	68.57
**CTOR15**	IMRT	Contour‐based	**55.07**	**59.59**	65.88
**CTOR16**	IMRT	Plan	22.22	16.5	42.84
**CTOR16**	IMRT	Contour‐based	29.97	16.54	37.52
**CTOR17**	IMRT	Plan	39.45	32.1	68.27
**CTOR17**	IMRT	Contour‐based	52.02	49.85	64.06
**CTOR18**	IMRT	Plan	**69.15**	**92.04**	68.88
**CTOR18**	IMRT	Contour‐based	**68.98**	**93.01**	63.7
**CTOR19**	IMRT	Plan	27.59	18.15	67.42
**CTOR19**	IMRT	Contour‐based	27.59	17.83	65.31
**CTOR20**	IMRT	Plan	22.64	14.08	65.29
**CTOR20**	IMRT	Contour‐based	22.79	14.92	57.66
**CTOR21**	IMRT	Plan	40.40	35.56	68.3
**CTOR21**	IMRT	Contour‐based	34.40	24.59	53.22
**CTOR22**	IMRT	Plan	**65.31**	**84.96**	63.46
**CTOR22**	IMRT	Contour‐based	**58.26**	**69.71**	61.42
**CTOR23**	IMRT	Plan	40.84	31.62	**73.68**
**CTOR23**	IMRT	Contour‐based	53.10	49.29	54.17
**CTOR24**	IMRT	Plan	35.66	28.84	66.02
**CTOR24**	IMRT	Contour‐based	40.92	30.02	**70.34**
**CTOR25**	IMRT	Plan	43.43	41.87	**70.52**
**CTOR25**	IMRT	Contour‐based	52.06	52.43	55.54
**CTOR26**	IMRT	Plan	21.36	15.82	**70.47**
**CTOR26**	IMRT	Contour‐based	22.72	17.44	57.68
**CTOR27**	IMRT	Plan	47.28	37.37	55.11
**CTOR27**	IMRT	Contour‐based	51.00	46.99	51.15
**CTOR28**	IMRT	Plan	21.33	16.08	67.69
**CTOR28**	IMRT	Contour‐based	27.47	19.99	53.2
**CTOR29**	IMRT	Plan	33.83	19.11	58.77
**CTOR29**	IMRT	Contour‐based	35.33	17.93	58.34
**CTOR30**	IMRT	Plan	46.11	14.05	47.87
**CTOR30**	IMRT	Contour‐based	46.75	14.33	45.89
**CTOR31**	IMRT	Plan	**56.87**	**60.03**	**75.72**
**CTOR31**	IMRT	Contour‐based	**58.08**	**62.29**	**78.89**
**CTOR32**	IMRT	Plan	48.99	**55.09**	60.89
**CTOR32**	IMRT	Contour‐based	51.26	**61.9**	63.38
**CTOR33**	IMRT	Plan	34.05	29.2	57.07
**CTOR33**	IMRT	Contour‐based	29.09	21.69	**76.88**
**CTOR34**	IMRT	Plan	47.14	39.88	66.1
**CTOR34**	IMRT	Contour‐based	51.42	48.43	59.47
**CTOR35**	IMRT	Plan	31.33	22.77	65.98
**CTOR35**	IMRT	Contour‐based	30.76	12.05	39.14
**CTOR36**	IMRT	Plan	50.71	50.47	57.76
**CTOR36**	IMRT	Contour‐based	52.31	52.34	60.03
**CTOR37**	IMRT	Plan	51.03	51.98	52.73
**CTOR37**	IMRT	Contour‐based	53.99	**68.79**	52.32
**CTOR38**	IMRT	Plan	51.51	41.43	61.65
**CTOR38**	IMRT	Contour‐based	51.66	42.33	65.5
**CTOR39**	IMRT	Plan	53.13	50.94	64.87
**CTOR39**	IMRT	Contour‐based	40.1	29.63	63.24
**CTOR40**	IMRT	Plan	46.03	44.09	**74.45**
**CTOR40**	IMRT	Contour‐based	**58.7**	**70.31**	**71.76**
**CTOR41**	IMRT	Plan	27.13	24.64	68.62
**CTOR41**	IMRT	Contour‐based	13.65	7.03	65.84
**CTOR42**	IMRT	Plan	35.92	0	44.5
**CTOR42**	IMRT	Contour‐based	40.54	0	42.04
**CTOR43**	IMRT	Plan	15.5	9.05	57.53
**CTOR43**	IMRT	Contour‐based	18.99	8.1	62.09
**CTOR44**	IMRT	Plan	49.41	50.09	57.04
**CTOR44**	IMRT	Contour‐based	50.62	52.79	48.14
**CTOR45**	IMRT	Plan	**72.08**	**94.5**	**72.04**
**CTOR45**	IMRT	Contour‐based	**70.61**	**93.91**	67.09
**CTOR46**	IMRT	Plan	**57.03**	**60.13**	69.43
**CTOR46**	IMRT	Contour‐based	**60.21**	**66.52**	61.51
**Total exceeds plan**	n/a	n/a	37	32	15
**Total exceeds delivered dose**	n/a	n/a	22	26	14
